# Interconnections of Hypoxia and Aging in Long-Lived Species

**DOI:** 10.1093/geroni/igaf122.2882

**Published:** 2025-12-31

**Authors:** Damla Yalcin, Hsin-Yu Fang, Erdogan Oguzhan Akyildiz, Sudenaz Fatma Oner, Anil Sukru Dogan, Thomas Park, Perinur Bozaykut Eker

**Affiliations:** Acıbadem Mehmet Ali Aydınlar University, Ataşehir/İstanbul, Istanbul, Turkey; Tufts University, Boston, Massachusetts, United States; Acıbadem Mehmet Ali Aydınlar University, Ataşehir/İstanbul, Istanbul, Turkey; Acıbadem Mehmet Ali Aydınlar University, Istanbul, Istanbul, Turkey; Bogazici University, Beşiktaş, Istanbul, Turkey; University of Illinois Chicago, Chicago, Illinois, United States; Tufts University, Boston, Massachusetts, United States

## Abstract

Hypoxia is traditionally considered harmful to cells, yet emerging evidence suggests it may promote longevity through unclear mechanisms. Two evolutionarily distant but ecologically similar species, the naked mole-rat (NMR) and blind mole-rat (BMR), exhibit exceptional longevity and hypoxia tolerance, making them ideal models for studying the molecular links between hypoxia and lifespan. Our previous work indicated that hypoxia is associated with several longevity interventions like calorie restriction at the transcriptomic level. This study explored whether hypoxia tolerance pathways overlap with longevity mechanisms, particularly within the nutrient-sensing axis. To investigate, we exposed NMRs, BMRs, and mice to normoxia and severe hypoxia (0% O_2_ for species-specific survival times) and assessed survival rates. We analyzed AMPK/mTOR signaling, autophagy, mitochondrial function, and inflammation in heart tissue, alongside metabolomics and oxidative phosphorylation (OXPHOS) assays. Our results demonstrated that long-lived species survived hypoxia significantly better than mice. Notably, enhanced AMPK signaling, elevated autophagy-related protein levels, and reduced oxidative stress in these species suggest a strong link between longevity and hypoxia resilience. Moreover, long-lived species preserved mitochondrial health under hypoxia, likely contributing to their extended lifespan. These findings highlight hypoxia’s potential role in longevity and suggest new therapeutic avenues for age-related diseases.

